# Coaches’ Motivational Style and Athletes’ Fear of Failure

**DOI:** 10.3390/ijerph16091563

**Published:** 2019-05-04

**Authors:** Juan Antonio Moreno-Murcia, Elisa Huéscar Hernández, Luis Conte Marín, Juan L. Nuñez

**Affiliations:** 1 Sports Research Center, Miguel Hernández University of Elche, Avda. de la Universidad, s/n, 03130 Elche, Spain; 2 Departament of Health Psychology, Miguel Hernández University of Elche, Avda. Universidad, s/n, 30202 Elche, Spain; ehuescar@umh.es; 3 Departament of Physical Activity and Sports, University of Murcia, C/Argentina, s/n, San Javier, 30720 Murcia, Spain; conte@um.es; 4 Department of Psychology, Sociology and Social Work, University of Las Palmas de Gran Canaria, C/Santa Juana de Arco, 1, 35004 Las Palmas de Gran Canaria, Spain; juanluis.nunez@ulpgc.es

**Keywords:** fear, autonomy support, controlling style, self-determined motivation, sport

## Abstract

*Background*: The purpose of this study was to examine the relationship between coaches’ interpersonal style and fear of failure in athletes. *Methods*: A sample of 340 athletes at the Federation Level with a mean age of 18.96 years (*SD* = 5.69 years.) comprised the sample. Athletes completed questionnaires related to fear of failure in sports as well as their perceptions of the extent to which their coaches provided support for athlete autonomy and control. *Results*: The results revealed a significant and positive relationship between coaches’ controlling style and athletes’ fear of failure whereas coach autonomy support was associated with reduced fear of failure. Through the use of cluster analysis, two athlete profiles emerged. One profile indicated moderate levels of fear of failure among those athletes who perceived a controlling coaching style. The second profile revealed a cluster of athletes with low levels of fear of failure and favorable perceptions of coach support for athlete autonomy. *Conclusions*: These findings provide further evidence for the role of coaches as social influences capable of contributing to both adaptive and maladaptive psychological outcomes for athletes in sports.

## 1. Introduction

Since the origins of Self-Determination Theory (SDT) [1,2,3] an extensive knowledge base has been generated about the influence of SDT variables on motivational and psychological processes in sport [4,5,6]. An important component of the theory pertains to the role of social agents in contributing to the psychological needs satisfaction of individuals in achievement contexts, which will enable favorable psychological, motivational, and emotional outcomes. Within the sport context, we know that coaches are extremely important social agents and that coaches’ interpersonal style plays an important role in contributing to the group’s motivational climate, social relationships and decision-making characteristics [7]. When the coach behavior facilitates autonomy support to the athletes, it is more likely that they have higher levels of perceived competence, autonomy, relatedness, and intrinsic motivation and they experience fewer maladaptive outcomes, such as avoidance, frustration, fear of failure, and burnout [8,9,10].

It is also recognized that when athletes engage in extensive social comparison and define success as “doing better than others” that they can tend to perceive competitive circumstances as stressful and to correspondingly experience higher customary levels of anxiety and fear of failure in these circumstances [11]. These unfavorable perceptions can also be stimulated by negative outcomes during their sport practice which, in turn, can result in committing additional mistakes. The anticipation of incurring a negative evaluation of important socialization agents, including from coaches, peers, and family members, can augment unfavorable expectations [12,13]. An additional complication can occur when coaches display a controlling interpersonal style, which is characterized by a behavior that fails to satisfy basic psychological needs and in which the coach displays frequent bouts of frustration and/or anger. In such circumstances, maladaptive psychological outcomes can result for athletes [8]. Tsai and Chen [14] noted that when the coach does not admit the mistakes of the athletes and they make use of the punishment, it causes them an increased fear of failing due to the pressure transmitted to achieve success.

Moreno-Murcia et al. [13] consider fear to correspond with a feeling or mood state in which the person interprets an environmental stimulus as alarming, or potentially dangerous, and this interpretation carries with it negative behavioral consequences. According to Conroy et al. [15], the mechanism by which fear of failure develops in athletes is through the perception that failure is possible, or eventual, and this fear is further enhanced when individuals have the perception that failure will bring adverse consequences [16]. One of the immediate consequences of this negative expectation is that the athlete perceives themself to be less competent in their sport and, as a defense mechanism, the person desires to avoid similar achievement situations [17]. Sport-related research has found that fear of failure can adversely impact the athlete’s motivation and unleash a pattern of responses that impede goal attainment [18,19]. Gustafsson, Sagar, and Stenling [20] have also found that a high level of fear of failure is related with burnout and psychological stress in track and field athletes. 

Bartholomew et al. [21] conducted a study in the physical education context in which a controlling teaching style was found to be associated with a fear of failure in adolescent students and was explained as well by the frustration of basic psychological needs. Coach provision of autonomy support has been found to predict basic psychological need satisfaction and to increase autonomous motivation in athletes [8]. Nonetheless, few studies have contemplated controlling coaching styles and fear of failure in athletes. Consequently, the purpose of this investigation was to examine the relationship between coaches’ interpersonal styles and athlete fear of failure characteristics. To identify groups of athletes who share similar responses in the fear-to-fail construct, a cluster analysis was carried out. The purpose of this analysis was to identify homogeneous groups or clusters based on their common characteristics. It was proposed that one profile with the athletes with a greater fear of failure in their sport, who would report that their coaches had more controlling interpersonal styles and a second profile with those athletes with lesser fear of failure who would participate under the guidance of coaches who provided greater autonomy support.

## 2. Materials and Methods 

### 2.1. Ethics Statement

This study has been approved by the Research Ethics Committee of Universidad Miguel Hernández de Elche (Elche, Spain) (DPS.JMM.01.17) and meets all ethical and legal standards that are applicable to the research of this survey modality.

### 2.2. Participants

The sample was comprised of 340 athletes at the Spanish Federation Level who had a mean age of 18.96 years (*SD* = 5.69) and with an age range of 13 to 59 years. The sample was randomly selected and included 210 males and 130 females. 279 of these athletes competed in individual sports (judo, track and field, swimming, artistic gymnastics, badminton, taekwondo, etc.) with the remaining 61 athletes being team sport athletes (basketball, soccer, among others).

### 2.3. Measures

#### 2.3.1. Fear of Failure 

The Performance Failure Appraisal Inventory-Revised (PFAI-R) [15] was used to assess fear of failure. The instrument has been translated and validated in the Spanish language and cultural context by Moreno-Murcia and Conte [22]. The questionnaire consists of 25 items and includes five subscales. The subscales (with corresponding sample questions) are labeled Fear of Experiencing Shame and Embarrassment (seven items: “When I am not succeeding, I am less valuable than when I succeed”); Fear of Devaluing One’s Self-Estimate (four items: “When I am failing, it is often because I am not smart enough to perform successfully”); Fear of Having an Uncertain Future (four items: “When I am failing, my future seems uncertain”; Fear of Important Others Losing Interest (five items: “When I am not succeeding, people are less interested in me”); and Fear of Upsetting Important Others (five items: “When I am failing, it upsets important others”). A Likert-type scale is used with responses ranging from “1” (“Do not believe at all”) to “5” (“Believe 100% of the time”). Internal consistency for the subscales ranged from 0.64 to 0.84 in this study. 

#### 2.3.2. Autonomy Support 

The Autonomy Support Scale. The ASS-PE [23] was employed in this study and consists of eleven items, which assess athletes’ perceived autonomy support from their coaches. The stem for the items is, “In my sport, my coach(es)…” and a sample question is “Help(s) us to understand the purpose of the activities in which we are involved”. A five-point Likert-type scale response format is used with endpoints of “1” (Definitely not true”) to “5” (Definitely true”). The Cronbach alpha index of internal consistency for the scale was 0.80 in this study.

#### 2.3.3. Controlling Style

The Controlling Style Scale. The CSS-PE [23] was used in the study and consists of nine items that assess athletes’ perceptions of a controlling coaching style. The stem for each item is, “In my sport, my coach…” and a sample item is, “Talks constantly and doesn’t allow us to provide input during practice”. A five-point Likert-type scale response format is used with this instrument with endpoints of “1” (Definitely not true”) to “5” (Definitely true”). The Cronbach alpha index of internal consistency was 0.65 in this study.

### 2.4. Procedure

Once the formal approval was obtained by the Ethics Committee of the principal investigator’s university, the second step in the study involved making contact with the coaches who would potentially be involved in the study and to explain the purpose of the study to them and, subsequently, to request that their athletes could be involved in the study through the completion of the questionnaires during athletes’ free time. A member of the research team provided each participant with written information about the study and an informed consent form. Parental permission was also requested from the parents of the younger athletes in order that their child could participate. Questionnaire completion occurred in the sport clubs and in various high-performance sport centers in Spain under the supervision of the lead investigator who explained how to complete the questionnaires and the researcher also proactively addressed any issues or questions that may have arisen at this time. All questionnaires were completed anonymously and typically required about fifteen minutes to complete. The data were collected in the months of March and April 2017. The response rate was 89% out of 382 questionnaires in total, of which 42 were rejected due to missing data in the response scales.

### 2.5. Data Analysis

Descriptive data (means and standard deviations) and bivariate correlations were obtained and Cronbach alpha values were also computed to assess the internal consistency for each of the subscales in the first phase of the data analysis. Table 1 provides the relevant descriptive information. Subsequently, two similarly sized samples of athletes were generated through random assignment. A hierarchical cluster analysis using Ward’s method with Sample 1 was then performed to determine whether common profiles existed within the sample related to patterns of fear of failure and coach interpersonal style. The same approach was then taken with Sample 2 to determine the extent to which there was consistency in the findings across Sample 1 and Sample 2. A hierarchical cluster analysis using Ward’s method was then used with the entire sample. In order to understand the characteristics of each motivational profile, and in relation to athlete performance assessments, a differential analysis was subsequently conducted at the final step. All data was analyzed using the SPSS 25.0 statistical package (IBM, Armonk, NY).

## 3. Results

### 3.1. Descriptive Statistics

The descriptive data revealed that the fear of failure scores were highest for the athletes on the Fear of Experiencing Shame and Embarrassment dimension, followed by Fear of Devaluing One’s Self-Estimate, Fear of Having an Uncertain Future, Fear of Important Others Losing Interest, and Fear of Upsetting Important Others. Coach autonomy support levels were relatively higher than coach controlling behaviors in this sample and significant negative correlations were found between coach autonomy support and each of the five subcategories on the Fear of Failure (PFAI) scale. Each of the fear of failure dimensions also was significantly correlated with each of the other fear of failure dimensions (Table 1) as well as with the coach controlling style. The coach autonomy support and coach controlling style were also significantly and negatively correlated.

### 3.2. Cluster Analysis

**Figure 1:**
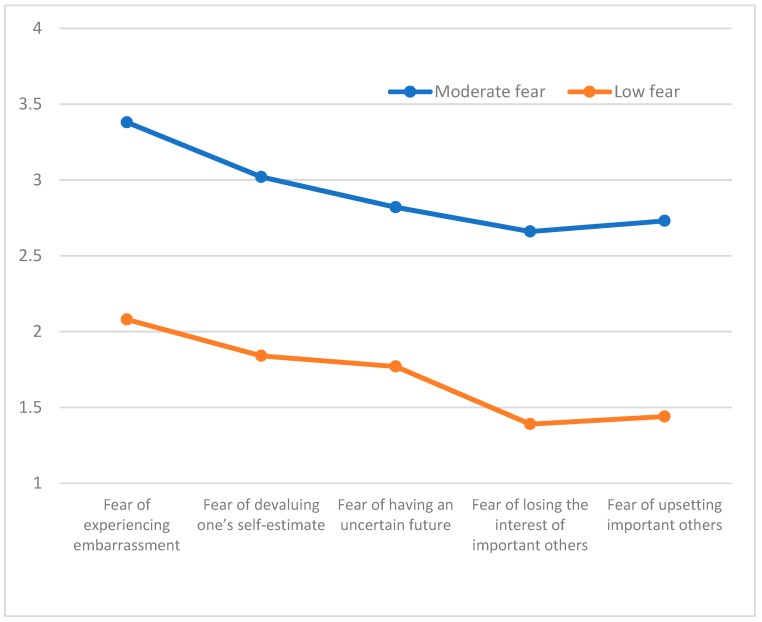
Profiles of Fear of Failure in Sample 1.

**Figure 2:**
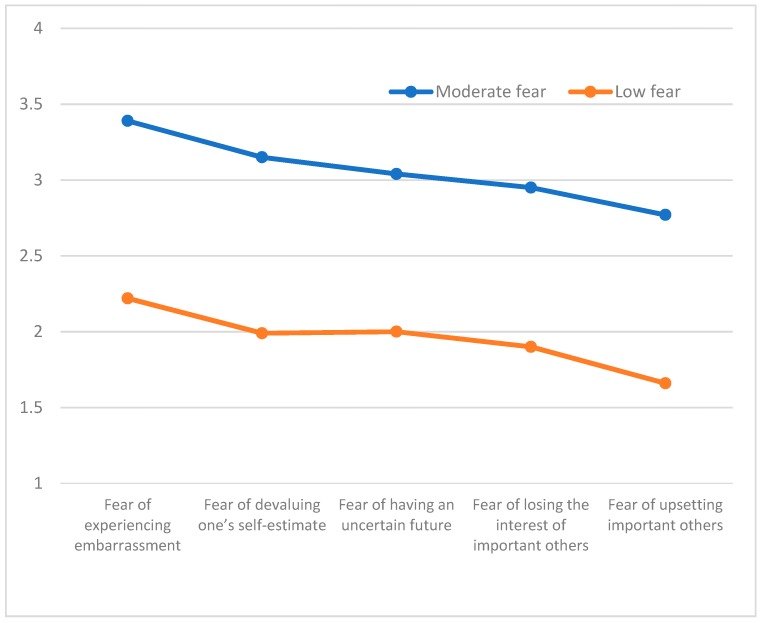
Fear of Failure profiles in Sample 2.

**Figure 3:**
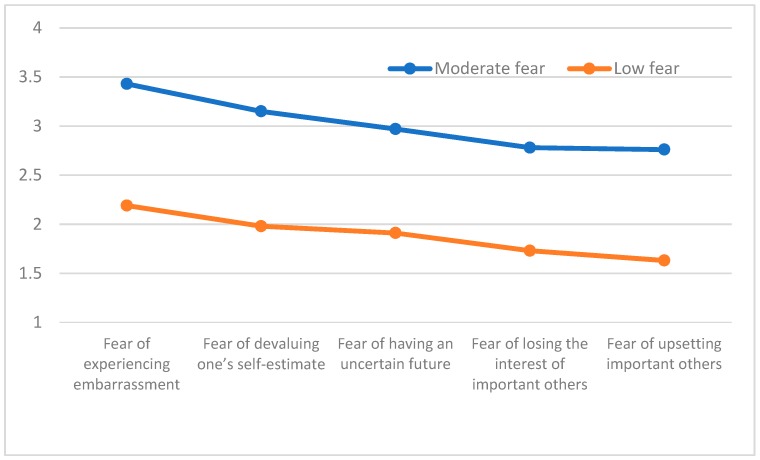
Fear of Failure profile in overall sample.

A cluster analysis was conducted in accordance with the stages proposed by Hair, Anderson, Tatham and Black [24]. The overall sample was separated into two equal samples through a randomization process. An agglomerative hierarchical approach was taken with the first sample (*n* = 169, M age = 18.39 yrs., *SD* = 6.85 yrs.) using Ward’s method. In order to determine the adequacy of the number of group classifications that emerged, the agglomeration coefficients were examined. As noted by Norusis [25] smaller coefficients indicate greater homogeneity among members of a given cluster whereas larger coefficients indicate greater differences among cluster members. The obtained dendogram indicated the existence of two groups that consisted of 75 and 94 individuals, respectively. Figure 1 contains representations of the “moderate fear” profile (Cluster 1) with moderate values associated with each of the fear of failure dimensions Cluster 2 was labeled “low fear” with relatively lower associated values across each of the fear of failure dimensions. Significant differences were identified between the two clusters, Wilks Λ = 0.26, *F* (5, 162) = 73.63, *p* < 0.001, η^2^ = 0.73 and these findings are presented in Table 2. Differences existed between profile groups on each of the fear of failure dimensions, including fear of experiencing embarrassment: *F* (1167) = 116.16, *p* < 0.001, η^2^ = 0.41; fear of devaluing one’s self-estimate, *F* (1167) = 143.60, *p* < 0.001, η^2^ = 0.46; fear of having an uncertain future *F* (1167) = 104.65, *p* < 0.001, η^2^ = 0.39, fear of losing the interest of important others, *F* (1167) = 166.49, *p* < 0.001, η^2^ = 0.50, and fear of upsetting important others, *F* (1167) = 188.71, *p* < 0.001, η^2^ = 0.53. To identify the characteristics of the group profiles in Sample 2 (Figure 2) (*n* = 170, *M* age = 17.97, *SD* = 4.05) a K-means test was employed to examine the groups consisting of 80 and 90 athletes, respectively (Table 2). A “moderate fear” (Cluster 1) profile emerged with values near the midpoint of each of the subscales and a second group, labeled “low fear” (Cluster 2) had low scale values on each of the five fear of failure subscales. A significant multivariate overall group difference was identified, Wilks Λ = 0.30, *F* (5164) = 76.54, *p* < 0.001, η^2^ = 0.70. Significant differences also were found for mean subscale values on each of the subscales, including: Fear of experiencing shame and embarrassment, *F* (1168) = 126.23, *p* < 0.001, η^2^ = 0.43; fear of devaluing one’s self-estimate, *F* (1168) = 126.40, *p* < 0.001, η^2^ = 0.43; fear of having an uncertain future, *F* (1168) = 128.84, *p* < 0.001, η^2^ = 0.43; fear of losing the interest of important others, *F* (1168) = 88.39, *p* < 0.001, η^2^ = 0.35; and fear of upsetting important others, *F* (1168) = 116.24, *p* < 0.001, η^2^ = 0.41. An agglomerative hierarchical analysis was subsequently conducted using Ward’s method with the entire sample which consisted of 210 and 130 athletes, respectively in the “moderate fear” and “low fear” profile groups (Figure 3). The results were similar to the previously obtained findings with significant differences between the groups, Wilks Λ = 0.35, *F* (5, 334) = 122.26, *p* < 0.001, η^2^ = 0.64 and on each of the individual fear of failure dimensions: Fear of experiencing shame and embarrassment, *F* (1338) = 227.21, *p* < 0.001, η^2^ = 0.40, fear of devaluing one’s self-estimate, *F* (1338) = 252.73, *p* < 0.001, η^2^ = 0.43; fear of having an uncertain future, *F* (1338) = 255.82, *p* < 0.001, η^2^ = 0.43; fear of losing the interest of important others, *F* (1338) = 162.06, *p* < 0.001, η^2^ = 0.33; and fear of upsetting important others, *F* (1338) = 241.71, *p* < 0.001, η^2^ = 0.42.

### 3.3. Differential Analysis

To examine possible differences between the fear of failure profiles in relation to athletes’ perspectives of coaching style, a differential analysis was conducted in which the clusters served as independent variables and the coaching styles as outcome variables. To determine whether the cluster profiles could be differentiated based upon the coach’s interpersonal style scores, a test of similarity of group means was conducted, Wilks Λ = 0.91, *F* (2337) = 15.97, *p* < 0.001, η^2^ = 0.09. These results revealed differences in autonomy support, *F* (1338) = 12.63, *p* < 0.01, η^2^ = 04 among the two clusters and indicated greater coach autonomy support in the “low fear” cluster (*M* = 3.78, *SD* = 0.61) relative to the “moderate fear” cluster (*M* = 3.54, *SD* = 0.58). As anticipated, there were significant differences between the two clusters in their mean scores on the perceptions of the coach’s controlling style, *F* (1338) = 28.39, *p* < 0.01, η^2^ = 0.08, with stronger perceptions of coach control in the “moderate fear” clusters, (*M* = 2.68, *SD* = 0.61) than in the “low fear” cluster (*M* = 2.32, *SD* = 0.58).

## 4. Discussion

From the standpoint of the Self-Determination Theory, it would be anticipated that coaches who adopt a motivationally-appropriate interpersonal style would contribute to the likelihood that their athletes will adopt more favorable motivational processes [9,21]. In accordance with this belief, the present study was designed to examine the relationship between coaches’ controlling or autonomy-supportive styles and athletes’ fear of failure through an examination of athlete profiles. The results obtained supported our expectation that coaching styles that reflected high levels of control and limited support for athlete autonomy would be associated with an athlete profile characterized by high levels of fear of failure. In fact, the cluster analysis revealed two distinct clusters in which one was characterized by low fear of failure and high levels of perceived autonomy support whereas the other reflected high levels of perceived coach control and moderate levels of fear of failure on behalf of athletes. 

These findings provide additional evidence of the importance of social agents in contributing to the psychological outcomes that athletes experience in sport and are thus consistent with some previous research [26,27]. More specifically, and in accordance with Bartholomew et al. [21], these findings provide an improved understanding of the influence of the coach’s interpersonal style on motivational and well-being characteristics. As such, results from the correlational analyses indicate that autonomy support was negative associated with fear of failure with greater support linked to lower fear. This finding is consistent with a body of knowledge that indicates that psychological nutriments increased well-being in athletes [28,29] whereas interpersonal styles heavy on coercive and controlling behaviors contribute to reduced well-being [21,30]. As such, autonomy-supportive and controlling behaviors on behalf of coaches exert contrasting effects even though the two dimensions of coach behavior are not necessarily polar opposites [31]. 

Consequently, the results of this study bring to light the role of coaches’ interpersonal styles in relation to athletes’ fear of failure. Coaches who continually use external incentives, threats, pressure and punishment can provoke frustration as well as a lack of well-being and burnout in their athletes [32,33] which can strengthen athletes’ fear of failure [34]. Conversely, coaches who help to shape their athletes’ orientation in sport through an emphasis on personal progression and improvement in which athletes have the capacity to make decisions can result in a reduction in fear of failure in sport. Another recent study with a similar sample of Federation Level basketball players [35] found that positive motivational climates initiated by coaches were associated with reduced fear of failure in athletes. 

Regarding the limitations of the study it should be mentioned that the investigation used cluster analysis, which reflects an exploratory and correlational foundation to understand a given phenomenon. As such, the effect sizes obtained through the analysis of differences are not particularly large overall. It would be useful to conduct further studies along this line of research that contrasted additional groups of athletes. In addition, with respect to the adjustment values of each measurement, not all factors have obtained a recommended internal consistency of 0.70 [36], but given the small number of items that make up the subfactor, the observed internal consistency can be marginally accepted [24,37]. In future investigations it would also be worthwhile to examine these outcomes in relation to gender, type of sport and in relation to age. In conclusion, pedagogical approaches that can enhance athlete autonomy and reduce the fear of failure associated with low competence perceptions should be beneficial. According to Moreno-Murcia and Sánchez [38], an appropriate autonomy-supportive environment can be characterized by a relaxed coach-athlete communication that is informal and flexible but not controlling; which is receptive to the opinions and input of athletes; includes active displays of empathy when listening; is oriented toward strengthening intrinsic motivation; regularly introduces novel and interesting activities; and allows the athlete sufficient time to accomplish the necessary tasks in an independent and autonomous way, with a focus on learning and not solely performance. An adaptive interpersonal style can thus proactively contribute to a reduction in some of the fear and avoidance behaviors that could otherwise occur in athletes and strengthen productive and healthy practices that contribute to greater long-term success and life satisfaction [39,40].

## 5. Conclusions

In closing, this study has revealed a significant and positive relationship between coaches’ controlling style and athletes’ fear of failure. In addition, two athlete profiles have emerged. One profile has indicated moderate levels of fear of failure among those athletes who perceived a controlling coaching style. The second profile has revealed a cluster of athletes with low levels of fear of failure and favorable perceptions of coach support for athlete autonomy. Therefore, the findings from this study can be presumed to be beneficial in the design and implementation of strategies that can minimize negative psychological outcomes that can occur in sports through controlling interpersonal behaviors of coaches [41,42]. Such approaches can contribute to greater individual and collective well-being and enhance the functioning of group members such that they are more likely to achieve the positive outcomes that are desired through their sport participation.

## Figures and Tables

**Table 1:** Means, standard deviations and correlations among the variables.

Variable	*M*	*SD*	α	R	1	2	3	4	5	6	7
1. Autonomy support	3.69	0.61	0.80	1–5	-	−0.36 **	−0.16 **	−0.13 *	−0.16 **	−0.23 **	−0.20 **
2. Coach controlling style	2.46	0.61	0.65	1–5	-	-	0.18 *	0.12 *	0.15 **	0.33 **	0.29 **
3. Fear of shame/embarrassment	2.66	0.95	0.84	1–5	-	-	-	0.64 **	0.51 **	0.48 **	0.53 **
4. Fear of devaluing one’s self-estimate	2.43	0.88	0.66	1–5	-	-	-	-	0.59 **	0.42 **	0.51 **
5. Fear of an uncertain future	2.31	0.79	0.64	1–5	-	-	-	-	-	0.48 **	0.54 **
6. Fear of important others losing interest	2.13	0.89	0.82	1–5	-	-	-	-	-	-	0.57 **
7. Fear of upsetting important others	2.07	0.85	0.78	1–5	-	-	-	-	-	-	-

Note: *p* < 0.05 *; *p* < 0.01 **.

**Table 2:** Standardized values, means and standard deviations of the variables in each cluster for Samples 1 and 2 and overall sample.

Variables	Sample 1				Sample 2				Sample Total			
	Cluster 1 (*n* = 75) Moderate Fear		Cluster 2 (*n* = 94) Low Fear		Cluster 1 (*n* = 80) Moderate Fear		Cluster 2 (*n* = 90) Low Fear		Cluster 1 (*n* = 210) Moderate Fear		Cluster 2 (*n* = 130) Low Fear	
	*M*	*SD*	*M*	*SD*	*M*	*SD*	*M*	*SD*	*M*	*SD*	*M*	*SD*
1. Fear of experiencing embarrassment	3.38	0.71	2.08	0.82	3.39	0.63	2.22	0.71	3.43	0.62	2.19	0.80
2. Fear of devaluing one’s self-estimate	3.02	0.64	1.84	0.63	3.15	0.73	1.99	0.62	3.15	0.65	1.98	0.67
3. Fear of having an uncertain future	2.82	0.80	1.77	0.52	3.04	0.52	2.00	0.66	2.97	0.60	1.91	0.59
4. Fear of losing the interest of important others	2.66	0.82	1.39	0.45	2.95	0.79	1.90	0.66	2.78	0.84	1.73	0.66
5. Fear of upsetting important others	2.73	0.72	1.44	0.49	2.77	0.77	1.66	0.57	2.76	0.73	1.63	0.60
